# High-Performance Blue Quasi-2D Perovskite Light-Emitting Diodes via Balanced Carrier Confinement and Transfer

**DOI:** 10.1007/s40820-022-00807-7

**Published:** 2022-02-23

**Authors:** Zhenwei Ren, Jiayun Sun, Jiahao Yu, Xiangtian Xiao, Zhaojin Wang, Ruijia Zhang, Kai Wang, Rui Chen, Yu Chen, Wallace C. H. Choy

**Affiliations:** 1 Department of Electrical and Electronic Engineering, The University of Hong Kong, Pokfulam Road, Hong Kong, People’s Republic of China; 2 Department of Electrical and Electronic Engineering, Southern University of Science and Technology, Shenzhen, 518055 People’s Republic of China; 3 School of Optoelectronic Science and Engineering, Soochow University, Suzhou, 215006 People’s Republic of China; 4 Guangdong-Hong Kong-Macao Joint Laboratory for Photonic-Thermal-Electrical Energy Materials and Devices, Shenzhen, 518055 People’s Republic of China

**Keywords:** Blue perovskite LEDs, Carrier confinement and transfer, Defect passivation, 2-Dimentional

## Abstract

Extensive investigation of the passivating agents has been performed to suppress the perovskite defects. However, very few attentions have been paid to rationally design the passivating agents for the balance of the carrier confinement and transfer in quasi-2D perovskites, which is essential to achieve high-performance perovskite LEDs (PeLEDs). In this work, tributylphosphine oxide (TBPO) with moderate carbon chain length is demonstrated as a decent passivator for the quasi-2D perovskites by strengthening the carrier confinement for massive radiative recombination within the perovskites, and more importantly providing efficient carrier transfer in the quasi-2D perovskites. Benefiting from these interesting optoelectronic properties of TBPO-incorporated perovskites, we achieve high-efficient blue PeLEDs with an external quantum efficiency up to 11.5% and operational stability as long as 41.1 min without any shift of the electroluminescence spectra. Consequently, this work contributes an effective approach to promote the carrier confinement and transfer for high-performance and stable blue PeLEDs.

**Supplementary Information:**

The online version contains supplementary material available at 10.1007/s40820-022-00807-7.

## Highlights

- A new concept to optimize the passivating agents for the balance of the carrier confinement and transfer in blue quasi-2D perovskites.
- The moderate tributylphosphine oxide is introduced into the quasi-2D perovskites to simultaneously strengthen the carrier confinement for massive radiative recombination as well as promote efficient carrier transfer in the quasi-2D perovskites.
- High-efficient and stable blue PeLEDs are achieved with an external quantum efficiency of 11.5% and a very long operational stability of 41.1 min without any shift of the electroluminescence spectra.

**Supplementary Information:**

The online version contains supplementary material available at 10.1007/s40820-022-00807-7.

## Graphical abstract

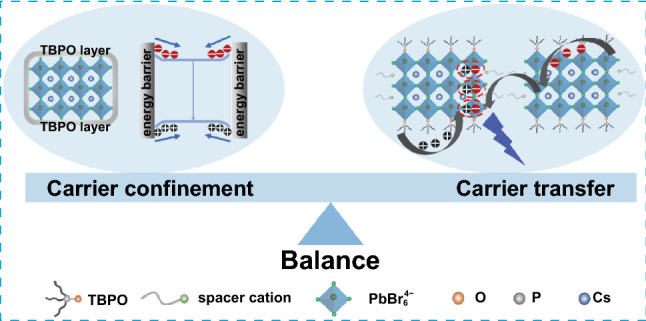

## Introduction

The excellent properties of metal halide perovskites, such as high color purity, tunable emission color, high photoluminescence quantum yields (PLQYs), and solution processability, promote the perovskites to become a competitive candidate for the next-generation light-emitting diodes (LEDs) [1–6]. Inspiringly, great progresses have been achieved in green, red, and near-infrared perovskite LEDs (PeLEDs) where impressive external quantum efficiencies (EQEs) exceeding 20% are reported [7–9], showing a great potential toward practical application. Although substantial efforts have been made in enhancing the blue PeLED performances, and efficiencies over 10% are achieved [10–14], the performances still lag far behind with the efficient green, red, and near-infrared PeLED analogs. The moderate blue PeLED performances undoubtedly retard the applications of PeLEDs in full-color displays and white-light illumination. It is highly desirable to develop highly performed and stable blue PeLEDs.

Recently, blue perovskite emitters, such as mixed-halide polycrystalline perovskites [11, 15–19], perovskite nanocrystals (NCs) [5, 10, 20–27], 2D perovskite nanoplatelets [28–32], and quasi-two-dimensional (quasi-2D) perovskites [33–41], have experienced extensive attention for efficient blue PeLEDs. Particularly, there is a booming development for the device efficiencies of quasi-2D perovskites taking the advantages of the energy funneling effect assisted fast exciton transfer and recombination, and the facilely solution-processed perovskite fabrication [42–44]. Typically, the component of quasi-2D perovskite consists of inorganic Pb–Br octahedral layer and organic spacer cation, which is generally named as B_2_(APbBr_3_)_*n−*1_PbBr_4_, where B is an organic spacer cation (e.g., phenylethylammonium: PEA^+^), A is a monovalent cation (e.g., Cs^+^, formamidinium: FA^+^), and *n* represents the number of Pb–Br octahedral layers [45, 46]. As we know, inevitable defects are generated during the crystallization of quasi-2D perovskites due to the ionic character of the hybrid perovskites. The defects are generally believed to be related to the ionic defects (e.g., halide vacancies) [47]. The halide vacancies can serve as nonradiative recombination centers, causing severe trap-mediated nonradiative losses and thus low emission efficiency. The vacancy defects can be effectively suppressed by molecular passivating agents which provide coordination or ionic bonding to neutralize the charged vacancies [48, 49]. For example, the molecules containing functional groups such as –NH_2_, C=O, or P=O can effectively passivate the vacancy defects of perovskite films by saturating the undercoordinated Pb sites through sharing the electron pairs with the empty orbital of undercoordinated Pb cations, thus contributing to high PLQYs for the perovskite films [48–50]. Specially, the phosphine oxide passivator of trioctylphosphine oxide (TOPO) was reported to boost the longest photoluminescence (PL) lifetime of polycrystalline perovskite films to over 8 microseconds which are comparable with the carrier lifetimes of single-crystal perovskite [51]. Meanwhile, TOPO was also reported to successfully passivate the green PEA_2_(FAPbBr_3_)_n−1_PbBr_4_ quasi-2D perovskite surface defects for bright emission and the subsequent efficient green PeLEDs [52], showing the great potential of phosphine oxide passivators for the preparation of high-performance PeLEDs.

Quasi-2D perovskites usually contain a mixture of layered perovskites with phase distribution from the low-*n* to high-*n* phases [45, 46]. This is because that there is a complex mixture of colloids with random size distribution in the precursor solution [53], which can act as nucleation centers to form 2D perovskites with different layers. In quasi-2D perovskites, the efficient energy transfer from the larger bandgap phase (i.e., lower-*n*) to the smaller bandgap phase (i.e., higher-*n*) is essential to realize the high emission efficiency [37, 52, 54]. Specially, different from the green-emissive quasi-2D perovskites, more organic spacer cations are needed to narrow the potential well of Pb–Br octahedral layers for blue emission [46, 55]. In other words, there are massive components of organic spacer cations in blue-emissive quasi-2D perovskites, which are harmful to the carrier transfer due to the insulating nature of the organic spacer cations [34, 55]. Meanwhile, there is a concern that the insulating carbon chain of phosphine oxide passivator will further aggravate the inefficient carrier transfer process; that is to say, the TOPO passivator may not be the ideal candidate for achieving efficient blue quasi-2D PeLEDs from its long carbon chain point of view. Consequently, it is rational and very important to optimize the passivating agents by promoting the carrier transfer in quasi-2D perovskites for high emission efficiency.

In this work, we systematically study the effects of phosphine oxide passivating agents of triethylphosphine oxide (TEPO), tributylphosphine oxide (TBPO), and trioctylphosphine oxide (TOPO) on the optoelectronic properties of quasi-2D perovskite films and demonstrate the TBPO-incorporated perovskites for the best-performed blue PeLEDs. Our results show that the appropriate carbon chain length of TBPO simultaneously offers good carrier confinement for the massive radiative recombination within the perovskites, and efficient carrier transfer in quasi-2D perovskites for high emission efficiency. Benefitting from the good optical and electrical properties of TBPO-incorporated perovskites, we achieve high-efficient blue PeLEDs with an EQE of 11.5% and prolonged operational stability of 41.1 min, which is among the best-performing blue PeLEDs. This work contributes an effective approach to design effective passivation molecules for high-quality perovskite films and efficient blue PeLEDs.

## Experimental Section

### Materials

Propylamine hydrobromide (PABr, 99.999%) and phenethylammonium hydrobromide (PEABr, 99.999%) were purchased from Greatcell Solar; cesium bromide (CsBr, 99.999%) and lead bromide (PbBr_2_, 99.999%) were bought from Sigma-Aldrich. Poly(9-vinylcarbazole) (PVK), tris(1-phenyl-1H-benzimidazol-2-yl)benzene (TPBi, 99.5%), and LiF (99.99%) were purchased from Lumtec; triethylphosphine oxide (TEPO, 98%), tributylphosphine oxide (TBPO, 98%), trioctylphosphine oxide (TOPO, 99%), and ethyl acetate (super dry, 99.9%) were purchased from J&K Chemical Ltd.

### Perovskite Emission Layer Preparation

The precursor of quasi-2D PEA_*x*_PA_2−*x*_(CsPbBr_3_)_*n*−1_PbBr_4_ perovskite was prepared by dissolving CsBr, PbBr_2_, PABr, and PEABr with a molar ratio of 1:1.1:1:0.2 into DMSO at a concentration of 0.15 mol L^−1^ (i.e., 0.15 M CsBr). Then, the pristine perovskite films were fabricated by dynamically spin-coating the precursor at 3000 rpm for 60 s. Specifically, 100 μL perovskite precursor was quickly dropped on the PVK substrate once the speed rate reaches 3000 rpm. Then, 300 μL of ethyl acetate was quickly dripped on the substrate at 26 s after the spin-procedure starting, followed by annealing at 70 °C for 10 min. For the phosphine oxides-incorporated perovskites, the preparation procedure is the same as the pristine perovskites, except for the antisolvent in which different concentrations of phosphine oxides were added.

### Device Fabrication

The indium tin oxide (ITO) substrates were successively washed with deionized water, acetone, and isopropyl alcohol and then further treated with oxygen plasma for 5 min before use. The PVK layer was prepared by spin-coating the PVK solution (4 mg mL^−1^ in chlorobenzene) on ITO substrates at 4000 rpm for 45 s and then heated at 120 °C for 15 min, according to our previous work [44]. Then, the perovskite precursor solution was spin-coated on PVK substrates to prepare the perovskite films. Finally, TPBi, LiF, and Al electrodes were successively evaporated onto the perovskite films with the thickness of 45, 1, and 90 nm with deposition rates of 0.5, 0.04, and 1 Å s^−1^, respectively. After that, the devices were sealed by an ultraviolet-curable resin before testing.

### Characterization

UV–Vis absorption spectra were carried out on the spectrophotometer of PERSEE TU-1901. The cross-sectional SEM characterization and the perovskite film morphologies were obtained using the scanning electron microscope (Zeiss 1550). The XPS measurements were performed with the use of Kratos Ultra Spectrometer equipped with monochromatized Al Kα X-ray photons discharge lamp. The depth-profiling XPS signals of the perovskite films were performed by etching the perovskite films with argon ions. The PLQYs were obtained using the equipment of Hamamatsu Quantaurus-QY, model No. C11347. TA spectra were obtained with an ExciPro XL Femtosecond Transient Absorption Pump-Probe Spectrometer (CDP systems). The perovskite films were pumped with a femtosecond 365 nm laser pulse generated from an optical parametric amplifier. The probe pulses with a range from 380 to 800 nm were generated from the fundamental 800 nm laser pulses by focusing a small portion (≈5 μJ) into a 2-mm-thick CaF_2_ plate. The PL and TRPL spectra of the perovskites were performed on PicoQaunt FluoTime 300 equipped with a picosecond pulse laser (360 nm, LDH-P-C-360). The capacitance–voltage (*C*–*V*) measurements were recorded using a Keithley 4200A parameter analyzer at 1 kHz with an AC amplitude of 20 mV. The capacitance–frequency (*C*–*f*) measurements were performed using Keithley 4200A parameter analyzer with the frequency range from 10^3^ to 10^5^ Hz and an AC amplitude of 25 mV. The value of absolute dielectric permittivity is 8.85 × 10^–12^ F m^−1^, the device area is 0.04 cm^2^, and the thickness of the perovskite film is 40 nm. The geometrical capacitances for pristine, pristine/TEPO, pristine/TBPO, and pristine/TOPO are 6.61, 5.79, 5.23, and 4.52 nF, corresponding to the calculated *ε* of 7.47, 6.54, 5.91, and 5.11, respectively. The current density–voltage–luminance curves were performed on Ocean Optics system equipped with a Keithley 2400 source meter, an integrating sphere (FOIS-1), and an QE Pro spectrometer. The integrating sphere was calibrated with a radiometric calibration light source (HL-3plus) before use. The active area for the devices is 4 mm^2^. The scanning rate is 0.1 V s^−1^ with a dwell time of 0.5 s. The CIE coordinates of the devices were measured on LED Color Calculator (OSRAM).

## Results and Discussion

**Fig. 1:**
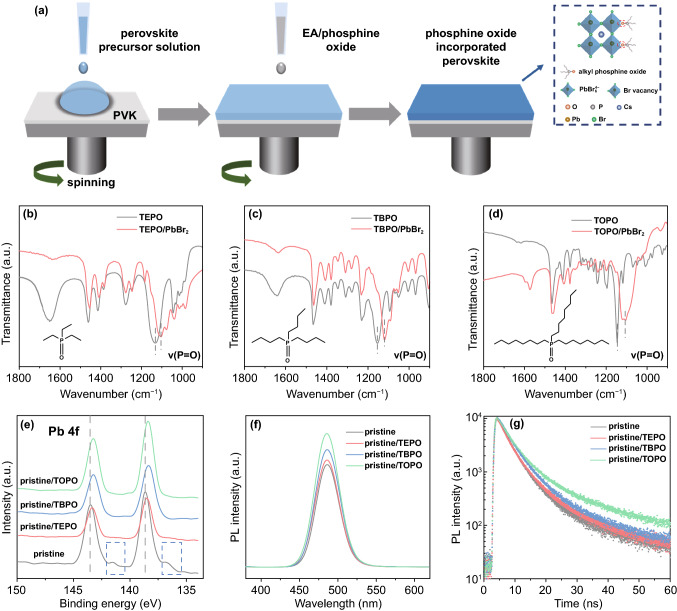
**a** Schematic illustration of the preparation of the perovskite films. FT-IR spectroscopy and the chemical structure of **b** TEPO, **c** TBPO, and **d** TOPO and their incorporated PbBr_2_ powder. **e** XPS spectra of Pb 4*f* signals, **f** steady-state photoluminescence, and **g** the time-resolved photoluminescence (TRPL) decay spectra of pristine and TEPO-, TBPO-, and TOPO-incorporated perovskite films

Quasi-2D perovskites with mixed spacer cations have been demonstrated to be capable of fine modulation of the perovskite phase for efficient blue PeLEDs [35, 56, 57]. Herein, the quasi-2D perovskite of PEA_*x*_PA_2−*x*_(CsPbBr_3_)_*n−*1_PbBr_4_ (PEA: phenylethylammonium, PA: propylammonium) is employed as the pristine perovskite due to the suppressed low-*n* perovskite phases for fast energy transfer and thus decent blue PeLEDs as shown in our previous works [56]. The pristine perovskite was prepared by spin-coating the perovskite precursor on PVK substrate followed by an antisolvent process with ethyl acetate (EA). For the passivating agents of alkyl phosphine oxides-treated perovskite films, EA with triethylphosphine oxide (TEPO), tributylphosphine oxide (TBPO), or trioctylphosphine oxide (TOPO) as an antisolvent was dropped on the perovskite films during the antisolvent process as illustrated in Fig. 1a. Details are shown in the experimental section.

### Interactions Between the Phosphine Oxides and the Perovskites

In TEPO, TBPO, and TOPO molecules, the P=O functional group provides the electron pairs to coordinate the unsaturated Pb^2+^ of perovskite films for halide vacancy passivation. The interaction between P=O and Pb^2+^ can be vividly verified by the Fourier-transform infrared spectroscopy (FT-IR) performed on TEPO, TBPO, TOPO, and their incorporated PbBr_2_ powder (abbreviated as TEPO/PbBr_2_, TBPO/PbBr_2_, and TOPO/PbBr_2_). As shown in Fig. 1b–d, the characteristic P=O stretching vibration peaks are located at 1132, 1153, and 1147 cm^−1^ for TEPO, TBPO, and TOPO [50, 52, 58], which shift to the lower wavenumbers to be 1106, 1118, and 1105 cm^−1^ for TEPO/PbBr_2_, TBPO/PbBr_2_, and TOPO/PbBr_2_, respectively. The shift of P=O stretching vibration to that lower frequency reveals a decreased electron cloud density between P and O atoms due to the complexation between P=O and PbBr_2_ through the oxygen atom of P=O with the empty 6p orbital of Pb^2+^. In addition, X-ray photoelectron spectroscopy (XPS) was performed to clarify the interaction of the phosphine oxides with the Pb–Br framework of perovskite films. As presented in Fig. 1e, the peaks centered at 143.5 and 138.6 eV for the pristine perovskite film are assigned to Pb 4*f* signal, which shift toward the lower binding energy upon incorporation of TEPO (pristine/TBPO), TBPO (pristine/TBPO), and TOPO (pristine/TOPO). The reduced binding energy indicates the lowered oxidation state of lead owing to the electron donation from the oxygen of the phosphine oxides. In addition, the similar shifts of Pb 4*f* peaks to the lower binding energy are observed when the pristine/TEPO, pristine/TBPO, and pristine/TOPO perovskite films are etched to a depth of 30 nm by argon ion during XPS measurement as described in experimental section, suggesting the permeation of TEPO, TBPO, and TOPO into perovskite films with the antisolvent treatment (Supplementary Fig. S1). Besides, two extra peaks at 141.1 and 136.2 eV are observed in pristine perovskites, which are attributed to the signals of metallic lead [59]. As reported previously, the presence of metallic lead is associated with halide vacancies in the perovskite lattice [59, 60], while no metallic lead signal is detected in pristine/TEPO, pristine/TBPO, and pristine/TOPO perovskites, indicating that the halide vacancies are effectively suppressed. Accordingly, it is found that the pristine/TEPO, pristine/TBPO, and pristine/TOPO perovskites show higher PL intensity than of pristine perovskite (Fig. 1f) with PLQYs increasing from 50.3% (pristine) to 56.4% (pristine/TEPO), 63.8% (pristine/TBPO), and 69.8% (pristine/TOPO), respectively. These indicate the reduction of nonradiative recombination upon the incorporation of TEPO, TBPO, and TOPO. In addition, the perovskite PLQYs increase in the order of pristine/TEPO, pristine/TBPO, and pristine/TOPO perovskites, showing that the longer carbon chain length of the phosphine oxides is more effective in suppressing the nonradiative recombination loss, which will be discussed in the following part. Consequently, we show that there is a strong interaction between the phosphine oxides and the perovskites and thus effectively suppress the halide vacancies for less nonradiative recombination.

### The Carrier Confinement Ability of the Phosphine Oxides

**Fig. 2:**
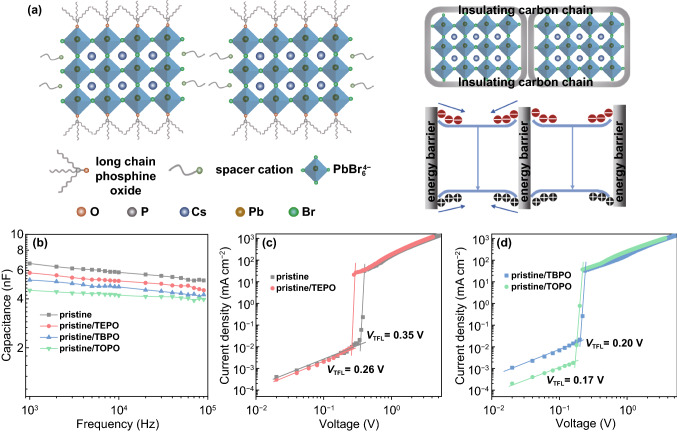
**a** Schematic illustration of the phosphine oxides on perovskite surfaces and the function of carbon chain for carrier confinement. **b** Capacitance–frequency (*C*–*f*) and **c**, **d** space charge limited current (SCLC) measurements of pristine, TEPO-, TBPO-, and TOPO-incorporated perovskite devices

To further study the effects of TEPO, TBPO, and TOPO on perovskite film carrier lifetime, the time-resolved PL (TRPL) measurements were performed on the phosphine oxides-incorporated perovskite films (Fig. 1g). The TRPL decay curves can be fitted by a biexponential function of *I* (t) = *A*_1_ exp(− *t*/*τ*_1_) + *A*_2_ exp(− *t*/*τ*_2_), where *A*_1_, *A*_2_ are the amplitudes, and *τ*_1_, *τ*_2_ are time constants for fast and slow decay components, respectively. The fast decay component is related to trap-assisted nonradiative recombination at grain surfaces, and the slow decay component can be attributed to the radiative recombination within the perovskites [6, 61]. The extracted parameters from the TRPL decay curves are recorded in Supplementary Table S1. It is revealed that the perovskite PL lifetimes gradually increase with the incorporation of TEPO, TBPO, and TOPO. The fast decay lifetimes are raised from 3.53 ns (pristine) to 3.82 (pristine/TEPO), 4.35 (pristine/TBPO), and 4.37 ns (pristine/TOPO), respectively, revealing the incorporation of phosphine oxides for reduced perovskite defects, consistent with the results of metallic lead suppression as shown in Fig. 1e. Meanwhile, the slow decay times greatly prolong from 12.81 ns (pristine) to 14.56 (pristine/TEPO), 19.82 (pristine/TBPO), and 22.08 ns (pristine/TOPO), respectively, which shows the strengthened radiative recombination within the phosphine oxides-incorporated perovskites. Notably, the slow decay times for pristine/TBPO and pristine/TOPO perovskites are much longer than pristine/TEPO perovskite, indicating the longer carbon chain of phosphine oxides for more radiative recombination within the perovskites. As illustrated in Fig. 2a, the phosphine oxides passivate the unsaturated Pb^2+^ through the coordination of the P=O functional group while leaving the carbon chain at the perovskite surfaces and/or boundaries. The longer carbon chains of TBPO and TOPO than TEPO act as an energy barrier which confines the carriers inside the perovskite due to the insulating nature and low permittivity of the carbon chain and thus reduces the nonradiative recombination for bright emission. In addition, it is reported that the carrier confinement ability of the organic chains can be evaluated by the dielectric mismatch between the organic chains and the inorganic layer (e.g., Pb–Br octahedral layer), and the smaller dielectric constant (*ε*) of the phosphine oxides will cause a larger dielectric mismatch for better carrier confinement [62]. Specifically, when decreasing the dielectric constant of the phosphine oxides, there is a weakened dielectric screening effect around the perovskites, which results in strong Coulomb force between electron and hole of the exciton for more radiative recombination (Supplementary Fig. S2). Therefore, the smaller dielectric constant of the phosphine oxides will contribute to better carrier confinement. To study the *ε* of the phosphine oxides, the capacitance–frequency (*C*–*f*) measurements were performed on the devices with a structure of ITO/PVK/perovskite/Al (Supplementary Fig. S3). The *ε* of the phosphine oxides can be compared from the *ε* of their incorporated perovskites according to the equation of *ε* = (*CL*)/(*ε*_0_*S*), where *C* is the geometrical capacitance, *L* is the thickness of the perovskite film, *ε*_0_ is the absolute dielectric permittivity, and *S* is the device area. As shown in Fig. 2b, the perovskite capacitance decreases after incorporation of TEPO, TBPO, and TOPO, indicating the lowered *ε* for the incorporated perovskite films. Furthermore, the calculated *ε* for pristine/TBPO and pristine/TOPO perovskites is 5.91 and 5.11, respectively, which are smaller than 7.47 and 6.54 of the pristine and pristine/TEPO perovskites. Since the dielectric constant of the perovskites changes depending on the incorporation of the phosphine oxides, the smaller *ε* of pristine/TBPO and pristine/TOPO perovskites implies the smaller *ε* for TBPO and TOPO and thus contributes to better carrier confinement. Besides, to rule out the effect of the perovskite defect concentration on the evaluation of the organic dielectric constant, the capacitance–frequency measurements were performed on the samples without perovskite precursors. The TBPO- and TOPO-incorporated films show lower capacitance than that of TEPO-incorporated one at the same preparation condition, indicating their smaller dielectric constants (Supplementary Fig. S4). In addition, the small *ε* values for pristine/TBPO and pristine/TOPO perovskites, together with the effective defect passivation roles of P=O function group, promote a small trap state density for the perovskites, which can be obtained from the space charge limited current (SCLC) measurements of the devices (ITO/perovskite/MoO_3_/Ag) by the equation of *N* = (2*V*_TFL_*ε*_r_*ε*_0_)/(*eL*^2^), where *N* represents the trap state density, *V*_TFL_ is the trap-filled limit voltage, and *ε*_r_ is the dielectric constant of the perovskites (Fig. 2c, d). The trap state density for pristine/TBPO and pristine/TOPO perovskites is 5.99 × 10^16^ and 8.16 × 10^16^ cm^−3^, respectively, which are much smaller than 1.81 × 10^17^ and 1.17 × 10^17^ cm^−3^ of the pristine and pristine/TEPO perovskites. Consequently, we show that the phosphine oxides can passivate the unsaturated Pb^2+^ of perovskite films through P=O functional group. Meanwhile, we demonstrate that the longer carbon chain of TBPO and TOPO than TEPO promotes more radiative recombination internal the perovskites due to the better carrier confinement induced by the insulating nature and low permittivity of the longer carbon chain. However, it is noted that the insulating nature of the carbon chain may also cause some problems (e.g., inefficient energy transfer in the quasi-2D perovskite, poor carrier injection, and transport in the PeLEDs), which will hinder the improvement of the device efficiency. Further characterizations of the optoelectronic properties for pristine/TBPO and pristine/TOPO perovskites will be studied in the following part.

### Energy Transfers in the Phosphine Oxides Incorporated Quasi-2D Perovskites

**Fig. 3:**
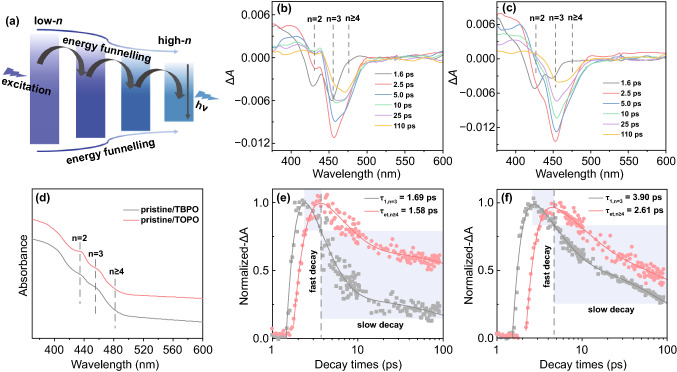
**a** Schematic illustration of the energy transfer process in quasi-2D perovskites. Transient absorption (TA) spectra of **b** pristine/TBPO and **c** pristine/TOPO perovskite films at different delay times. **d** The UV–visible absorption spectra of pristine/TBPO and pristine/TOPO perovskites. The extracted kinetics of the TA signals at selected wavelengths (453 and 480 nm) for **e** pristine/TBPO and **f** pristine/TOPO perovskite films

In quasi-2D perovskites, the photogenerated carriers are driven from a larger bandgap (i.e., low-*n*) phase to a smaller bandgap (i.e., high-*n*) phase and, subsequently, recombine in the high-*n* phase (Fig. 3a) due to the energy funneling effect [46]. Therefore, an efficient energy transfer from low-*n* phase to high-*n* phase in the quasi-2D perovskite is vital for high emission efficiency. To study the photogenerated carrier transfer dynamics in the pristine/TBPO and pristine/TOPO perovskites, the characterization of ultrafast transient absorption (TA) spectroscopy has been conducted. Figure 3b, c shows the TA spectra of pristine/TBPO and pristine/TOPO perovskites at different decay times, respectively. Three distinctive ground-state photobleaching (PB) signals can be observed at around 429, 453, and 480 nm, which are corresponding to *n* = 2, *n* = 3, and *n* ≥ 4 phases [35, 44]. The signals are consistent with their steady-state absorption spectra (Fig. 3d), where two shoulders of the absorption spectra centered at around 429 and 453 nm and a red-tail extending to ≈ 480 nm are observed. Besides, it is found that the photogenerated carriers are initially generated in low-order (i.e., *n* = 2, *n* = 3) phases with a fast buildup of *n* = 2 and *n* = 3 PB peaks in pristine/TBPO and pristine/TOPO perovskites. With increase in the decay time, the signals of low-order phases decrease. Meanwhile, the high-order (i.e., *n* ≥ 4) PB peak gradually increases which indicates the energy transfer from low-order phases to high-order ones [37]. To obtain more details of the carrier transfer process, we extract the time traces of low-order (e.g., *n* = 3, 453 nm) phase and high-order (e.g., *n* ≥ 4, 480 nm) phase from the decay kinetics (Fig. 3e, f). The kinetics of each PB can be fitted by a multiexponential function: ∆*A*(t) = *a*_1_ exp(− *t*/*τ*_1_) + *a*_2_ exp(− *t*/*τ*_2_) + *a*_3_ exp(− *t*/*τ*_3_) – *c*_1_ exp(− *t*/*τ*_et_), where *a*_1_, *a*_2_, *a*_3_, and *c*_1_ are amplitudes, *τ*_1_ represents the fast decay time constant due to the carrier transfer process between perovskite phases, *τ*_2_ and *τ*_3_ represent the slow decay time constant, and *τ*_et_ is the formation time constant obtained from the raising component of the PB signal [37, 63]. The fitting parameters are extracted in Supplementary Table S2. The PB of *n* = 3 phase shows a fast decay time with τ_1_ of 3.90 ps for pristine/TOPO perovskite, which closely matches with the *τ*_et_ (2.61 ps) of the *n* ≥ 4 phase PB (Fig. 3f), indicating a fast carrier transfer process from *n* = 3 phase to *n* ≥ 4 phase. However, the τ_1_ decreases to 1.69 ps for pristine/TBPO perovskite (Fig. 3e), which is less than half of pristine/TOPO perovskite (3.90 ps), indicating more efficient energy transfer from the low-order phase (*n* = 3) to the high-order phase (*n* ≥ 4). Meanwhile, the *τ*_et_ of *n* ≥ 4 phase for pristine/TBPO perovskite is 1.58 ps which is also smaller than that of pristine/TOPO perovskite (2.61 ps), further verifying the faster energy transfer process in pristine/TBPO perovskite. In consequence, the TA measurements show that pristine/TBPO perovskites have more efficient energy transfer from the low-order phase (*n* = 3) to the high-order phase (*n* ≥ 4) than that of pristine/TOPO perovskite, which is beneficial to high device performance as will be discussed later.

### Device Performances of the Phosphine Oxides Incorporated Blue PeLEDs

**Fig. 4:**
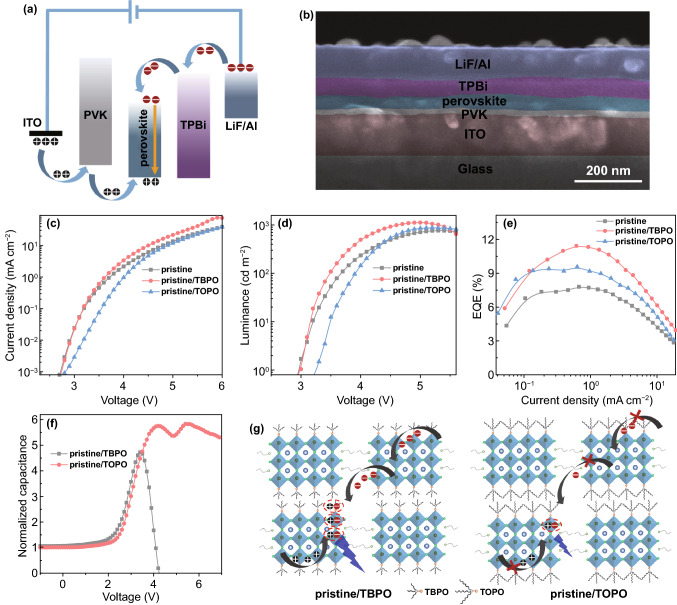
**a** Schematic illustration of the PeLED structure, **b** the cross section scanning electron microscopy (SEM) image of PeLEDs, and PeLED performance: **c** current density–voltage (*J*–*V*), **d** luminance–voltage (*L*–*V*), and **e** efficiency–current density (*EQE*–*J*) curves. **f** The device capacitance–voltage (*C*–*V*) measurements and **g** the illustration of charge transfer process in pristine/TBPO and pristine/TOPO PeLEDs

The PeLEDs were fabricated to evaluate the electroluminescence (EL) performances of the phosphine oxides-incorporated perovskites. The devices are assembled with the structure of ITO/PVK/perovskite/tris(1-phenyl-1H-benzimidazol-2-yl)benzene (TPBi)/LiF/Al, in which the perovskite emission layer is sandwiched between the hole injection layer (PVK) and the electron injection layer (TPBi) as illustrated in Fig. 4a. The cross-sectional scanning electron microscopy (SEM) image of the device is displayed in Fig. 4b. The thicknesses of PVK, perovskite, TPBi, and LiF/Al are around 20, 40, 45, and 90 nm, respectively. Meanwhile, the SEM images show uniform perovskite films with free pinholes for pristine/TBPO and pristine/TOPO perovskites (Supplementary Fig. S5).

The maximum luminance of pristine PeLEDs in Fig. 4d is 761 cd m^−2^ under the bias of 5.3 V, which increases to 868 and 1119 cd m^−2^ for pristine/TOPO and pristine/TBPO devices. Accordingly, the EQE and current efficiency (CE) are dramatically enhanced from 7.8% and 11.8 cd A^−1^ (pristine) to 9.6% and 15.7 cd A^−1^ (pristine/TOPO), and 11.5% and 17.7 cd A^−1^ (pristine/TBPO), respectively (Fig. 4e; Supplementary S6). The improved pristine/TOPO and pristine/TBPO device performances are further verified by the decent average EQEs which increase from 6.9% (pristine) to 8.3% (pristine/TOPO) and 10.4% (pristine/TBPO), respectively, extracted from 15 devices for each condition (Supplementary Fig. S7). The improvements can be attributed to the effective defect passivation and carrier confinement of TBPO and TOPO for massive radiative recombination. Importantly, the pristine/TBPO PeLEDs show higher efficiency than pristine/TOPO devices, benefitting from the more efficient energy transfer from the low-order phase to the high-order phase in pristine/TBPO perovskite, as shown in TA spectra (Fig. 3e). Furthermore, it is found that the pristine/TOPO PeLEDs exhibit smaller current density (Fig. 4c) and larger turn-on voltage (corresponding to a luminance of 1 cd m^−2^) (Fig. 4d) than that of pristine/TBPO PeLEDs, which reveal a larger carrier injection barrier for pristine/TOPO PeLEDs and thus show only moderate improvement of the device efficiency. To further verify the charge injection and recombination capacity of pristine/TBPO and pristine/TOPO PeLEDs, the capacitance–voltage (*C*–*V*) measurements of the devices were performed (Fig. 4f). It is observed that the capacitances of pristine/TBPO and pristine/TOPO devices show different trends against the bias voltages. In the *C*–*V* curves, the capacitance increases with the raise of the bias voltage, indicating that the charges are injected into the devices, while the capacitance of pristine/TBPO devices rises faster than that of pristine/TOPO devices, indicating more charges are injected. When further increasing the voltages, the capacitance of pristine/TBPO devices reaches the peak value and then sharply declines due to the largely reduced charges caused by the radiative recombination of massive electrons and holes [64]. Differently, the capacitance of pristine/TOPO devices drops slightly after the peak capacitance then rises again. This variation vividly illustrates the less efficient radiative recombination of holes and electrons in pristine/TOPO PeLEDs. The results well agree with the moderate brightness of 868 cd m^−2^ for pristine/TOPO devices, which is smaller than that of pristine/TBPO devices (1119 cd m^−2^). The decent role of TBPO for improved brightness can be further applied to the green PeLEDs (Supplementary Fig. S8), in which the pristine/TBPO PeLEDs show much higher brightness of 3411 cd m^−2^ than that of pristine PeLEDs (2135 cd m^−2^), indicating the general role of TBPO for high device performance. We also investigate the effect of different TBPO and TOPO concentrations on the PeLED performances (Supplementary Figs. S9 and S10), in which the highest efficiencies are obtained for 2 mM TBPO- and TOPO-incorporated PeLEDs. The performance will decline at a higher concentration. Particularly, the TOPO-incorporated PeLEDs show a large decrease in the efficiency with the increase in the concentration to 3 mM, which can be attributed to the enlarged injection barrier as evidenced by the increased turn-on voltage (Supplementary Fig. S10b). Besides, the pristine/TEPO PeLEDs are also fabricated and the efficiency distribution extracted from 15 devices is shown in Supplementary Fig. S11. The highest efficiency of pristine/TEPO PeLEDs is 8.9% with an average of 7.9% which is lower than that of pristine/TBPO (10.4%) and pristine/TOPO (8.3%) devices due to the weak carrier confinement as shown previously.

**Fig. 5:**
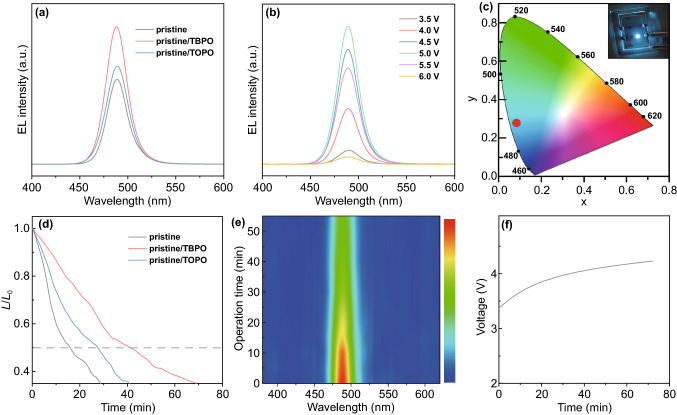
**a** EL spectra of pristine, pristine/TBPO, and pristine/TBPO PeLEDs. **b** EL spectra of pristine/TBPO PeLEDs at different bias voltages and **c** the CIE coordinate of pristine/TBPO PeLEDs with the emission image in the inset. **d** The time-dependent stability lifetime measurements under 0.4 mA cm^−2^ of pristine, pristine/TBPO, and pristine/TBPO PeLEDs. **e** The traced EL spectra of pristine/TBPO PeLEDs during operation and **f** the corresponding bias voltage traces during the lifetime test

The EL spectra of pristine, pristine/TOPO, and pristine/TBPO devices are centered at around 488 nm (Fig. 5a), which are consistent with their PL spectra, indicating that the EL is merely derived from the perovskites. Besides, the EL peak wavelength remains unchanged with the increase in bias voltage from 3.5 to 6.0 V (Fig. 5b; Supplementary S12) and thus achieves good spectral stability for the PeLEDs as verified by the neglectable variation of the corresponding Commission Internationale de L’Eclairage (CIE) coordinates (Supplementary Fig. S13, Table S3). Meanwhile, the pristine/TBPO PeLEDs show a narrowband emission with the full width at half maximum (FWHM) of around 25 nm, which enables a good color purity with CIE chromaticity coordinate at (0.078, 0.28) (Fig. 5c). Regarding operational stability, the lifetime of the PeLEDs was measured under a continuous constant current (0.4 mA cm^−2^) since LEDs are usually current driven devices. The lifetime (*T*_50_) defines as the working time of PeLED decaying to 50% of its initial luminance (*L*_0_). The lifetime for pristine/TBPO PeLEDs reaches 41.1 min at the initial luminance of 33 cd m^−2^ (Fig. 5d), which is longer than that of pristine (15.9 min, *L*_0_: 21 cd m^−2^) and pristine/TOPO (27.3 min, *L*_0_: 28 cd m^−2^) and represents one of the most stable blue PeLEDs [23, 25, 35, 37, 57, 65–71]. Meanwhile, the EL spectra of pristine/TBPO PeLEDs keep unchanged and exhibit excellent stability under continuous operation (Fig. 5e). It is observed that the bias voltage of pristine/TBPO PeLEDs increases with prolonging the operation time (Fig. 5f) due to the increased device resistance during operation. We also measured the device lifetime under a larger injection current of 1.2 mA cm^−2^ as shown in Supplementary Fig. S14. The lifetime for pristine/TBPO is 11.2 min at the initial luminance of 102 cd m^−2^, much longer than that of 5.4 and 8.1 min for pristine and pristine/TOPO PeLEDs. Consequently, our results show that the pristine/TBPO PeLEDs have excellent spectral stability and the longest lifetime, which promotes a great potential for the blue PeLEDs toward practical applications.

## Conclusion

In summary, we report to rationally study and optimize the passivating agents by controlling the carbon chain length to simultaneously enhance the carrier confinement for massive radiative recombination internal the perovskites and provide the efficient carrier transfer in quasi-2D perovskites. By further studying of the optoelectronic properties of the phosphine oxides (TEPO, TBPO, and TOPO)-incorporated perovskites, we demonstrate a good balance between carrier confinement and transfer for TBPO with the moderate chain length. TBPO offers better carrier confinement than TEPO and provides more efficient carrier transfer than TOPO. Based on the TBPO-incorporated perovskites, high-efficient blue PeLEDs with EQE up to 11.5% and operation lifetime as long as 41.1 min are achieved, which are among the best-performing blue PeLEDs reported so far. Consequently, our work provides an effective way for the realization of high-performance and stable blue PeLEDs.

## Supplementary Information

Below is the link to the electronic supplementary material.

Supplementary file1 (PDF 662 KB)
