# Supplementary material for: High-Performance Blue Quasi-2D Perovskite Light-Emitting Diodes via Balanced Carrier Confinement and Transfer
Source: Nanomicro Lett. 2022 Feb 23;14:66. doi: 10.1007/s40820-022-00807-7 (PMC8866581; doi:10.1007/s40820-022-00807-7)
Supplement: Supplementary file 1 — Supplementary file1 (PDF 662 KB) [file 40820_2022_807_MOESM1_ESM.pdf]

Supporting Information for

High-Performance Blue Quasi-2D Perovskite Light-Emitting Diodes *via* Balanced Carrier Confinement and Transfer

Zhenwei Ren[1, 3], Jiayun Sun[1], Jiahao Yu[2], Xiangtian Xiao[1], Zhaojin Wang[2], Ruijia Zhang[1], Kai Wang[2, *], Rui Chen[2, *], Yu Chen[3], Wallace C. H. Choy[1, 4, *]

[1]Department of Electrical and Electronic Engineering, The University of Hong Kong

Pokfulam Road, Hong Kong, P. R. China

[2]Department of Electrical and Electronic Engineering, Southern University of Science and Technology, Shenzhen 518055, P. R. China

[3]School of Optoelectronic Science and Engineering, Soochow University, Suzhou 215006, P. R. China

[4]Guangdong-Hong Kong-Macao Joint Laboratory for Photonic-Thermal-Electrical Energy Materials and Devices, Shenzhen 518055, P. R. China

*Corresponding authors. E-mail: wangk@sustc.edu.cn (Kai Wang), chenr@sustech.edu.cn (Rui Chen), chchoy@eee.hku.hk (Wallace C. H. Choy)

Supplementary Figures and Tables

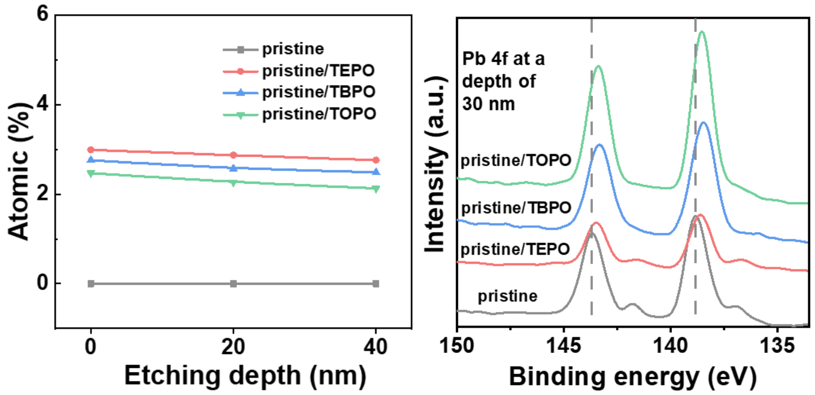

Fig. S1 (**a**) XPS spectra of Pb 4f signals at the depth of 30 nm in pristine and TEPO, TBPO, and TOPO incorporated perovskite films. (**b**) The atomic ratio of phosphorus atom for the perovskite films at different etching depths. The distributions of phosphorus atom at different etching depth show that the atomic ratios of phosphorus atom decrease 7.7%, 9.8%, and 13.7% at the etching depth of 40 nm for pristine/TEPO, pristine/TBPO, and pristine/TOPO perovskite films, respectively. The larger decrease of phosphorus atomic ratio for pristine/TBPO and pristine/TOPO perovskite films than that of pristine/TEPO indicates that the larger molecule size of TBPO and TOPO than that of TEPO can deteriorate the permeation of TBPO and TOPO to a certain degree

Table S1 The fitted parameters of TRPL decay curves based on pristine and phosphine oxides incorporated perovskite films with a biexponential function of I (t) = A_1 exp(−t/τ_1) + A_2 exp(−t/τ_2)

perovskites	A_1	τ_1 (ns)	A_2	τ_2 (ns)	$\tau_{average}$ (ns)
pristine	32448.21	3.53	1235.23	12.81	4.66
pristine/TEPO	29890.28	3.82	1167.31	14.56	5.21
pristine/TBPO	27247.78	4.35	738.75	19.82	6.05
pristine/TOPO	24754.36	4.37	1230.12	22.08	7.92

Table S2 The fitting parameters extracted from a multi-exponential function: $\Delta A(t) = a_1 \exp(-t/\tau_1) + a_2 \exp(-t/\tau_2) + a_3 \exp(-t/\tau_3) - c_1 \exp(-t/\tau_{et})$, where a_1, a_2, a_3, and c_1 are amplitudes; τ_1 is the fast decay time constant ascribed to the energy transfer process in the perovskite; τ_2 and τ_3 represent the slow decay constant, and τ_{et} is the formation time constant

Perovskites		τ_1/ps (a_1)	τ_2 /ps (a_2)	τ_3 /ps (a_3)	τ_{et} /ps (c_1)
pristine/TBPO	n=3	1.69 (2.69)	97.59 (0.21)	-	0.37 (71.25)
	n≥4	0.87 (6.87)	5.42 (0.77)	625.12 (0.64)	1.58 (22.77)
pristine/TOPO	n=3	3.90 (0.95)	76.74 (0.43)	-	0.47 (71.86)
	n≥4	8.53 (0.52)	61.55 (0.32)	2851.31 (0.38)	2.61 (38.58)

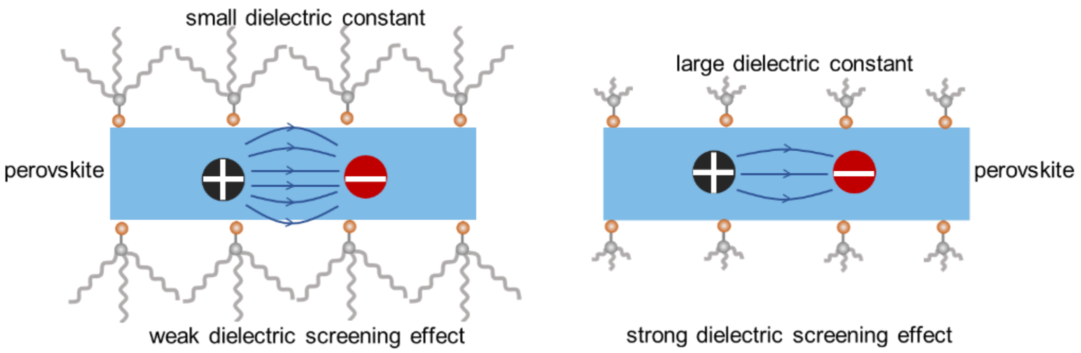

Fig. S2 Schematic illustration of the dielectric screening effects of organic molecules with small and large dielectric constant

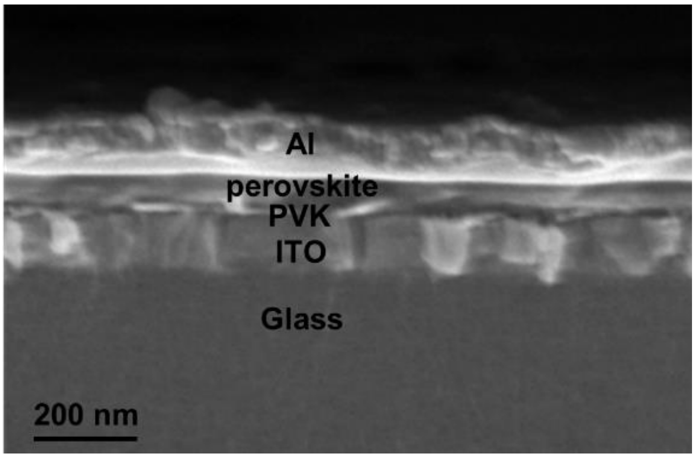

Fig. S3 Cross-sectional scanning electron microscopy (SEM) image of the device with a structure of ITO/PVK/perovskite/Al, in which the thicknesses of ITO, PVK, perovskite, and Al are around 103 nm, 20 nm, 40 nm, and 101 nm

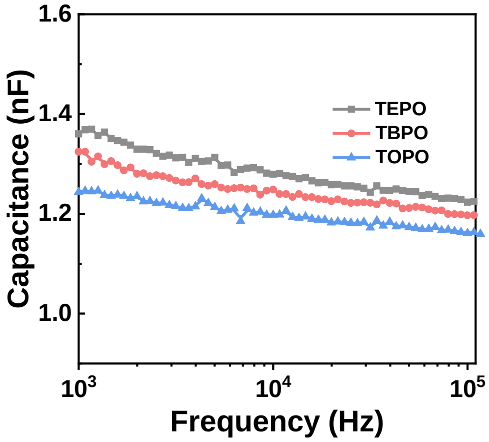

Fig. S4 Capacitance-frequency (*C-f*) measurements of TEPO, TBPO, and TOPO incorporated films without perovskite (CsBr, $PbBr_2$) components

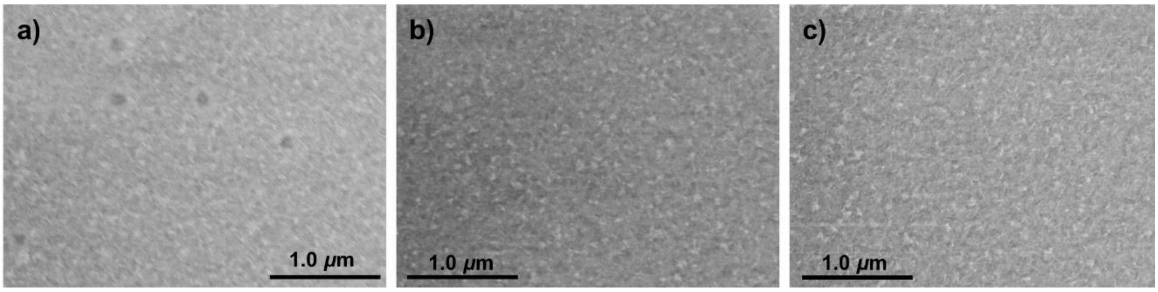

Fig. S5 The SEM images of (**a**) pristine, (**b**) pristine/TBPO, and (**c**) pristine/TOPO perovskite films

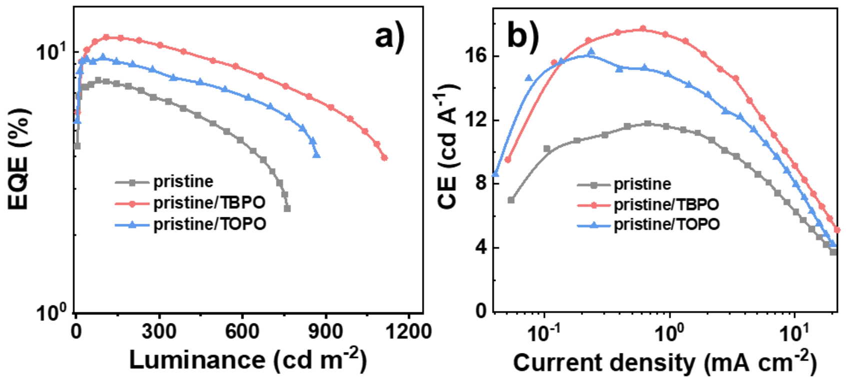

Fig. S6 The curves of (**a**) EQE–luminance (*EQE–L*) and (**b**) current efficiency–current for pristine, pristine/TBPO, and pristine/TOPO PeLEDs

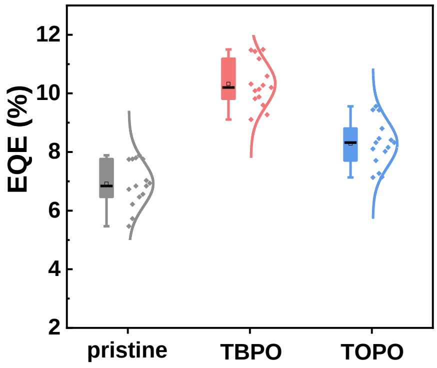

Fig. S7 Statistical graph of pristine, pristine/TBPO, and pristine/TOPO PeLEDs obtained from15 devices for each condition

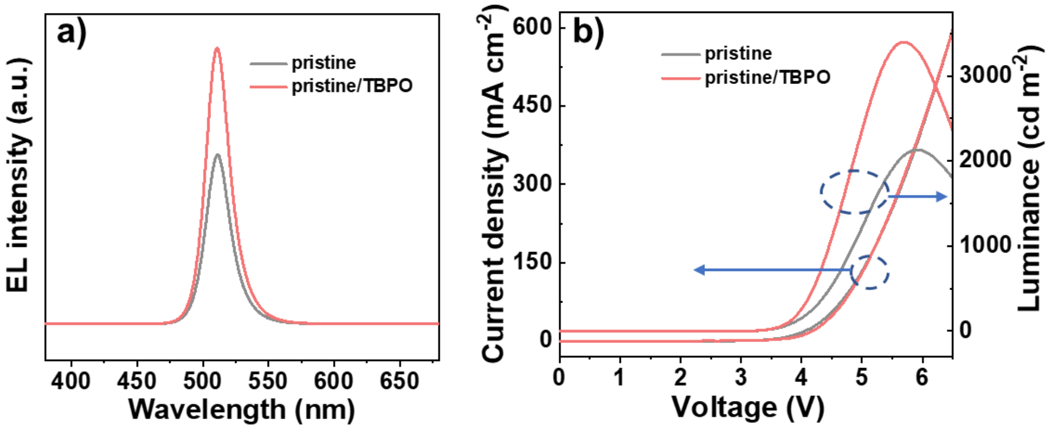

Fig. S8 (**a**) The electroluminescence (EL) spectra and (**b**) the current density–voltage-luminance (*J–V–L*) curves for pristine and pristine/TBPO green PeLEDs. The green PeLEDs were fabricated by spin-coating the perovskite precursor (CsBr: $PbBr_2$: PEABr = 1:1:0.4) on PVK substrate with similar method to that of blue PeLEDs in the manuscript

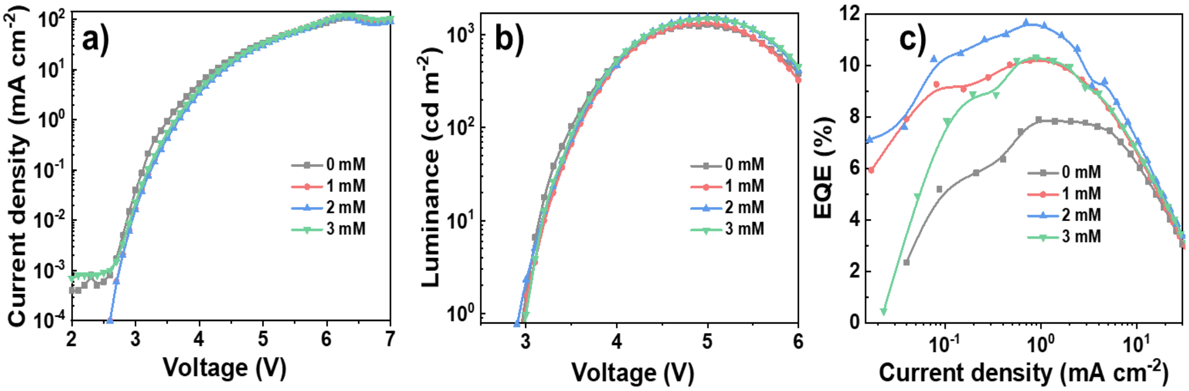

Fig. S9 The performances of pristine/TBPO PeLEDs with incorporation of different TBPO concentrations: (**a**) current density–voltage (*J–V*), (**b**) luminance–voltage (*L–V*), and (**c**) efficiency–current density (*EQE–J*) curves

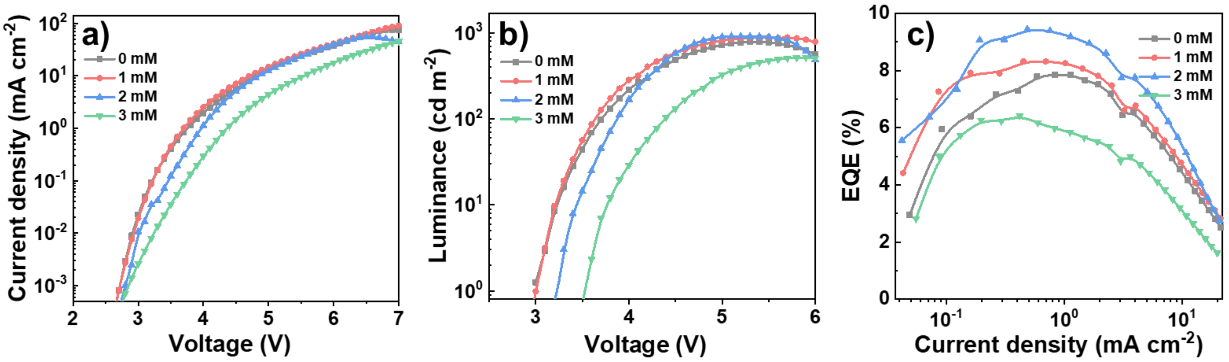

Fig. S10 The performances of pristine/TOPO PeLEDs with incorporation of different TOPO concentrations: (**a**) current density–voltage (*J–V*), (**b**) luminance–voltage (*L–V*), and (**c**) efficiency–current density (*EQE–J*) curves

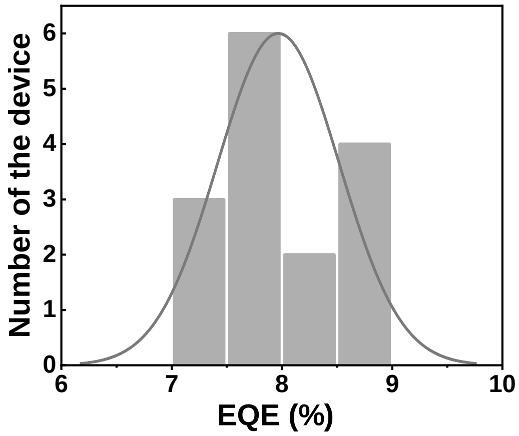

Fig. S11 Histograms of 15 devices for pristine/TEPO PeLEDs with the maximum efficiency of 8.9% and an average efficiency of 7.9%

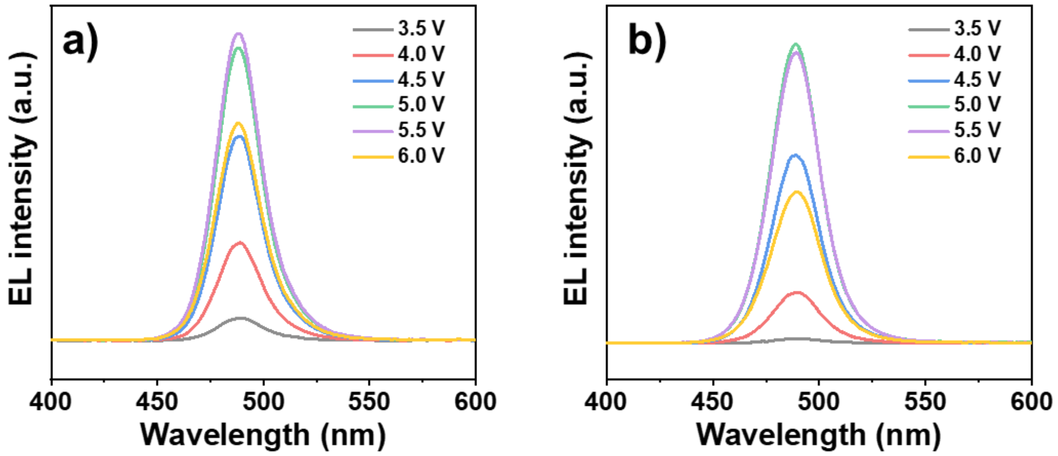

Fig. S12 EL spectra of (**a**) pristine and (**b**) pristine/TOPO PeLEDs at different bias voltages

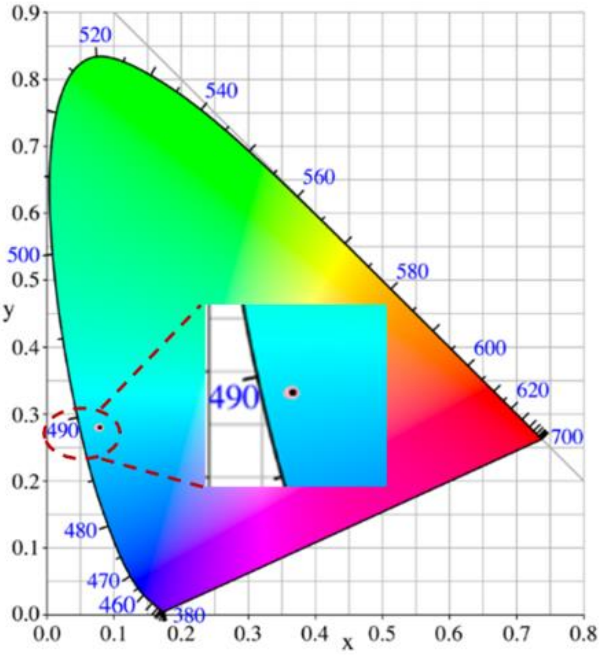

Fig. S13 CIE coordinates of pristine/TBPO PeLEDs with increase of the bias voltage from 3.5 to 6.0 V (the enlarged area is shown in the inset)

Table S3 CIE coordinates of pristine/TBPO PeLEDs during the stability test

Voltage (V)	x	y
3.5	0.076	0.28
4.0	0.076	0.28
4.5	0.077	0.28
5.0	0.078	0.28
5.5	0.078	0.28
6.0	0.079	0.28

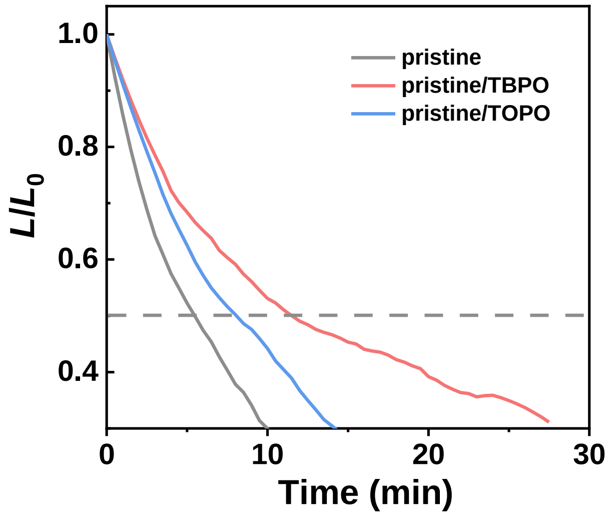

Fig. S14 The time-dependent stability lifetime measurements under 1.2 mA cm^{-2} of pristine, pristine/TBPO, and pristine/TOPO PeLEDs with the initial luminance of 83, 102, and 90 cd m^{-2}
